# Supplementary material for: Are carbon emissions trading and green financial instruments synergistic? -Comprehensive quantitative research based on content analysis
Source: PLoS One. 2024 Mar 7;19(3):e0298601. doi: 10.1371/journal.pone.0298601 (PMC10919601; doi:10.1371/journal.pone.0298601)
Supplement: S1 Data — (ZIP) [file pone.0298601.s001.zip › Data/地级市Z_F工作报告.zip!/╔╜╢1⁄2/╤╠╠¿╩╨/└┤╫╘╣1⁄2╓┌║┼í╛┬φ┐╦╩2╛▌═°í┐_2011.docx]

各位代表：

现在，我代表市人民政府向大会报告工作，请予审议，并请市政协委员和其他列席会议的同志提出意见。

一、“十一五”时期的政府工作

“十一五”时期是我市发展史上极不平凡的五年。面对复杂多变的国内外环境和前所未有的风险挑战，全市上下在中共烟台市委的正确领导下，坚持以邓小平理论和“三个代表”重要思想为指导，深入贯彻落实党的十七大和十七届三中、四中、五中全会精神，以科学发展观统领全局，解放思想、抢抓机遇，锐意进取、攻坚克难，加快转方式调结构步伐，全面推进改革开放和现代化建设，认真执行市人大及其常委会的各项决议，圆满完成了“十一五”规划目标任务，实现了经济社会发展的新跨越。

———过去的五年，是经济平稳较快发展、区域综合实力显著增强的五年。全市生产总值2008年迈上3000亿元台阶，2010年迈上4000亿元台阶，达到4358.5亿元，是2005年的2倍，年均增长14.9%；人均生产总值9396美元。境内税收收入完成515.2亿元，地方财政收入完成237.8亿元，年均分别增长22.2%和22.3%。全社会固定资产投资完成2705.9亿元，年均增长23.4%。社会消费品零售总额达到1378.4亿元，年均增长19.2%。到2010年末，全市存贷款余额分别达到4081.2亿元和2644.5亿元，比2005年底分别增加2398.2亿元和1496.5亿元。

———过去的五年，是产业结构优化升级、科学发展迈出坚实步伐的五年。一、二、三次产业比例由2005年的9.8:59.5:30.7调整为7.7:58.9:33.4。工业经济迅猛发展。机械、电子、食品、黄金等传统支柱产业持续扩张，汽车、手机、电脑、船舶、葡萄酒等产品集群快速膨胀，鸿富泰电子、南山集团、通用东岳汽车等一批骨干企业不断壮大。2010年，全市实现规模以上工业主营业务收入10967亿元，年均增长24.7%，利润、利税年均分别增长26.5%和24.9%。主营业务收入过百亿元企业由2005年的1家增加到12家，利税过亿元企业由60家增加到133家。中国名牌产品、中国驰名商标分别由12个和7件增加到28个和41件。高新技术产业产值占规模以上工业总产值的比重由28.6%提高到44.1%。入选国家创新型城市试点，引进了中科院海岸带研究所和中科院烟台综合技术转化中心，新增国家级工程技术研究中心2家、重点实验室1家、企业技术中心10家、创新型企业5家。五年累计申请专利26800件，取得科技成果965项，获国家科学技术奖17项、省科学技术奖214项，其中获国家科技进步一等奖1项。节能减排“十一五”目标任务全面完成，二氧化硫、化学需氧量排放量五年分别下降13.2%和19.4%，万元GDP能耗下降22%。服务业快速崛起。旅游会展、商贸餐饮、房地产等传统服务业发展良好，现代物流、文化创意、服务外包、金融保险等高端服务业蓬勃兴起。“十一五”时期，全市旅游总收入累计达到1164亿元，是“十五”期间的2.7倍。成功举办了国际葡萄酒节和国际果蔬·食品博览会等一系列重要节会。2010年，全市港口货物吞吐量达到2.1亿吨，集装箱吞吐量达到154.1万标箱，年均分别增长19.8%和17.3%。航空客运吞吐量和货邮吞吐量分别达到249.6万人次和4.3万吨，年均分别增长15.5%和19%。莱山国际机场开通至台北客运航线，至荷兰阿姆斯特丹和美国芝加哥、洛杉矶等国际货运航班。全市服务业增加值完成1457.5亿元，年均增长16.9%。现代农业发展质量进一步提高。粮食连续八年丰收，果品、蔬菜、畜牧、水产等优势行业稳步发展，农业产业化、标准化、国际化步伐加快，新增7件国家地理标志证明商标和5个国家地理标志保护产品。全面落实强农惠农政策，五年累计发放各类涉农补贴20.5亿元。

———过去的五年，是各项改革不断深化、对外开放取得丰硕成果的五年。国有资产监管体制不断完善，完成了冰轮、氨纶等171户企业改革改制，全市国有资产总量由2005年的138亿元增加到423亿元。新增私营企业1.5万家、个体工商户6.8万户，民营经济实现增加值、上缴税金分别占全市生产总值、国地税收入的80%和60%以上。新增上市公司14家，总数达到26家，五年累计募集资金184亿元。农村集体林权制度改革深入实施，医药卫生体制改革全面展开，新一轮政府机构改革顺利进行，财政体制改革、事业单位改革、乡镇机构改革稳步推进，金融、教育、文化、社会保障等各领域改革取得新进展。2010年，全市完成外贸进出口总额437.8亿美元，年均增长30.7%，占全省的比重由2005年的14.9%提高到23.2%。其中出口254.8亿美元、进口183亿美元，年均分别增长31.5%和29.6%。机电产品和高新技术产品出口比重分别由2005年的35%和15.9%提高到71.7%和43.3%。五年累计实际使用外资51亿美元，新引进世界500强企业和知名跨国公司30多家。“走出去”战略深入实施，五年累计实施境外投资项目112个，外派劳务2.2万人次。

———过去的五年，是城镇化步伐进一步加快、城乡面貌发生深刻变化的五年。编制完成了城市总体规划、海岸带规划和覆盖城乡的相关专项规划。中心城市五年累计完成城建投入600亿元以上，新建和改扩建红旗路、北马路、科技大道等30余条主次干道，打通魁星楼、黄金顶等4条隧道，高标准建设了体育公园、文化中心、火车站等标志性建筑，启动了大南山、夹河、辛安河“一山两河”和沿海一线生态景区建设，实施了迎春大街、幸福区片、毓璜顶医院周边等915万平方米旧城旧村改造项目。大力推进市区环境综合整治和农村生态环境建设，城市“洁绿亮美”水平不断提高，农村面貌明显改观。新增公交线路33条。城区融合和镇村建设步伐加快，中心城市建成区面积由172平方公里扩展到240平方公里，小城镇建成区面积达到206平方公里，建成新型农村社区1204处。全面启动农村住房建设和危房改造工程，建设农房16万户，改造危房2.2万户。全市城镇化率达到56.5%，比2005年提高3.9个百分点。努力突破交通瓶颈，烟大铁路轮渡建成运营，蓝烟铁路电气化改造、莱山国际机场改造等工程全面完成，烟台港西港区、潮水国际机场、青烟威荣城际铁路、龙烟铁路、烟台至海阳高速公路等一批重点工程开工建设。五年累计改修建干线公路1765公里，完成农村公路硬化里程4852公里。建成生活垃圾处理场9座，新建扩建污水处理厂11座，新增集中供热面积2870万平方米、天然气用户21万户。加强城市饮用水源地保护，完成胶东引黄调水工程进度目标，农村自来水普及率达到96.1%。全面完成“三年病险水库除险加固”和“三年大造林”任务，实施了16座大中型水库和621座小型水库除险加固工程，新增造林绿化面积69.7万亩，森林覆盖率达到40%。

———过去的五年，是民生工程扎实推进、各项社会事业全面进步的五年。2010年，城市居民人均可支配收入达到23288元，农民人均纯收入达到9916元，年均分别增长13.3%和13.1%。每年实施为民服务十件实事工程，解决了一批群众普遍关心的热点难点问题。五年新增城镇就业再就业74.2万人，“十一五”末，城镇登记失业率为3.25%。企业最低工资、企业退休人员基本养老金、老年农民养老待遇、城乡低保标准稳步提高。新型农村合作医疗实现全覆盖，城镇基本医疗保险覆盖率达到90%，农村社会养老保险累计参保240万人。五年累计提供经济适用房12632套，3900余户住房困难家庭享受到廉租房待遇。城市和农村居民人均住房面积分别由2005年的18.7平方米和27.7平方米增加到29.4平方米和35.4平方米。新建了烟台老年福利服务中心、儿童福利院和残疾人托养服务中心等一批公共服务设施。深入开展“爱心捐助”活动，累计接收爱心捐款7.8亿元，救助困难群众和困难学生105万人次。提高中小学生公用经费标准，全面实施标准化学校建设工程、校舍安全工程、农村中小学“211”工程及图书馆（室）建设。在全省、全国率先全部免除城乡义务教育阶段杂费和课本费，全市初中和高中段入学率均达到100%。市区社区卫生服务人口覆盖率达到97%，农村三级医疗卫生服务网络更加完善。新建1个国家级、6个省级文化产业示范基地，全市乡镇、街道全部建成国家规定标准的综合文化站。成功承办了第21届省运会、第7届省残运会和第11届全运会部分赛事，烟台籍运动员在北京奥运会、广州亚运会等国内外重大赛事上取得优异成绩。全民健身工程覆盖全市80%以上社区和90%以上规划布点村。帮扶栖霞、支持长岛和对口支援工作取得新成果。人口和计划生育工作保持先进水平，气象地震工作在防灾减灾中发挥了重要作用，老龄、妇女、儿童、红十字、残疾人和慈善事业得到加强，外事侨务、广播电视、新闻出版、史志档案、双拥共建、人民防空、民族宗教、国防后备力量建设等各项社会事业取得新进步。

———过去的五年，是民主法制日益加强、和谐稳定局面更加巩固的五年。基层群众自治机制逐步完善，社会法治化管理水平不断提高。五年累计办理市人大代表建议、市政协提案5600件次，办结率达到100%。坚持依法行政，认真落实政务公开和民主决策制度，推行行政执法责任制，扎实开展“改进机关作风、优化政务环境”活动，深化行政审批制度改革，行政工作效率和服务水平不断提升。加强信访稳定工作，高度重视市长公开电话、市长信箱建设，广泛听取民意，及时解决群众反映的问题。全面加强廉政建设，维护了政府机关和公务员队伍的良好形象。广泛开展创先争优、“读唱讲做”和群众性精神文明创建活动，城市公共文明指数明显提高。安全生产工作进一步加强，食品药品监管力度逐步加大，应急管理水平得到巩固提升。积极创建和谐稳定模范城市，认真做好群众工作，社会治安形势持续稳定。

2010年是实施“十一五”规划的最后一年，也是应对国际金融危机取得显著成效的一年。初步核算，全市生产总值增长14.1%；地方财政收入增长25.7%；固定资产投资增长22.8%；社会消费品零售总额增长18.8%；实际使用外资增长6.3%，外贸进出口总额增长27.7%；城市居民人均可支配收入增长10.2%，农民人均纯收入增长14.8%；万元GDP能耗、二氧化硫和化学需氧量排放均完成省下达目标。

各位代表，回顾过去五年，我们走过了非同寻常的发展历程。全市上下牢牢把握“昂扬精神迎挑战，科学务实谋发展”的工作主基调，直面挑战，加压奋进，经受了严峻考验，取得了重大成就，为全市长远发展赢得了主动、抢占了先机。面对国家新一轮区域战略调整，高起点实施山东半岛蓝色经济区、胶东半岛高端产业聚集区、黄河三角洲高效生态经济区“三大战略”，龙口湾、丁字湾、莱州湾等集中集约用海聚集区进入国家重点开发范围，总投资1320亿元的66个海洋重点产业项目启动建设。经济技术开发区综合实力在国家级园区中跃居第6位，高新区升级为国家高新技术产业开发区，保税港区获批设立并开关运作，一批拉动经济快速增长的新板块、新亮点正在形成。面对扩内需、保增长的重大政策机遇，迅速制定配套措施，积极对接项目和政策，深入开展“项目推进年”和“消费推动年”活动，争取中央和省预算内投资项目541个，落实扩内需资金35.7亿元。新机场、新港口、“三条铁路干线”等多年期盼的重大项目先后上马，化学工业园、张裕工业园、高端铝合金产业园和通用东岳汽车新增30万辆轿车、宝钢精密钢管等战略性产业项目陆续启动，海阳核电、华电国际莱州电厂等能源项目相继开工，实现了重大项目建设的历史性跨越。面对百年不遇的国际金融危机冲击，不畏艰难险阻，提振发展信心，破解发展难题，在行政审批、融资担保、鼓励出口、刺激消费、税费改革等方面先后出台了40多项帮扶政策，减轻企业负担48亿元。积极协调煤电油气运等要素保障，助推企业渡过难关。全市经济不仅率先企稳回升，而且在抵御危机的过程中积累了经验、积蓄了能量，迈出了突破发展的新步伐。面对自然灾害和突发事件等重重困难，保持清醒头脑，坚定必胜信念，万众一心、共克时艰，全力以赴支援汶川特大地震、青海玉树地震、甘肃舟曲泥石流等自然灾害抢险救灾工作，提前圆满完成对口援建北川任务，受到了灾区人民的广泛赞誉。群策群力、顽强抗击强降雪、风暴潮等自然灾害，创造了防雪抗灾的“烟台经验”。积极参与“4?28”胶济铁路事故应急处理，妥善处置问题奶粉、甲型H1N1流感、浒苔灾害、矿山事故、海上抢险等重大突发事件，圆满完成奥运安保任务，维护了和谐稳定的良好局面。

各位代表！经历“十一五”时期的发展与磨练，今天的烟台，经济更加繁荣，社会更加和谐，人民更加幸福，城市知名度和美誉度显著提高。五年来,我市获得或连续获得全国文明城市、全国社会治安综合治理优秀城市、中国投资环境金牌城市、全国双拥模范城、中国和谐管理城市、中国城市综合创新能力五十强、国家知识产权示范城市、中国食品名城、中国绿色食品城、联合国改善居住环境最佳范例奖以及中国最佳休闲城市等一大批荣誉。广大市民的自豪感和荣誉感不断增强，干事创业的热情空前高涨，对未来的发展充满信心。

五年的探索与实践使我们深深体会到，应对各种复杂局面和重大挑战，实现经济社会的跨越发展，必须始终坚持党的正确领导，认真贯彻上级党委、政府的各项决策部署，讲政治、顾大局，确保政令畅通；必须始终坚持科学发展，推进“五个统筹”，积极转方式、精心调结构，走全面、协调、可持续发展之路；必须始终坚持解放思想，主动适应形势变化，立足实际，趋利避害，化不利为有利，变挑战为机遇，锲而不舍攻坚克难，励精图治加快发展，在逆境中营造发展新优势；必须始终坚持走烟台特色发展道路，发挥自身优势，突破薄弱环节，举全市之力争取大政策、实施大战略、推进大项目，抢占发展制高点，在各个领域积极作为、奋勇争先；必须始终坚持改革开放，创新体制机制，破除影响科学发展的长期性、结构性矛盾，在更宽领域、更高层次开展国际交流与合作，全面激发加快发展的动力和活力；必须始终坚持以人为本，保障和改善民生，维护社会公平正义，让全市人民共享改革发展成果。

过去的五年，我们取得了令人瞩目的成就。这一切得益于党中央、国务院，省委、省政府和市委的正确领导，得益于历届政府打下的坚实基础，得益于市人大、市政协和社会各界对政府工作的有效监督与大力支持，得益于全市人民的团结奋斗、拼搏进取和无私奉献。在此，我代表市人民政府，向全市人民，向市人大代表、政协委员，向各民主党派、工商联、各人民团体和各界人士，向中央、省属驻烟单位，向驻烟解放军、武警官兵，致以崇高的敬意！向所有关心支持烟台发展的香港和澳门特别行政区同胞、台湾同胞、海外侨胞、国际友人，表示衷心的感谢！

在总结成绩的同时，我们也清醒地看到，我市经济社会发展还存在一些亟待解决的困难和问题，主要表现为：经济平稳运行面临复杂形势，资源环境的约束日益增强，经济发展的不确定因素增多；服务业发展相对滞后，自主创新能力还不够强，发展方式转型任务十分艰巨；民间投资和消费潜力还没有充分激活，扩大开放面临严峻挑战；财政收支矛盾依然突出，金融生态环境有待优化；区域发展不够平衡，部分居民的生活还比较困难，农村生产生活条件还需要继续改善；民生投入仍需加强，就业、住房、医疗、教育等与群众生活密切相关的热点难点问题解决得还不够好；部分机关工作人员作风不实、效率不高，腐败现象还时有发生，政府公共服务水平有待进一步提高，等等。对于这些问题，我们将采取有效措施，认真加以解决。

二、“十二五”时期的奋斗目标

“十二五”时期是我市全面建设小康社会的关键时期，是深化改革开放、加快转变经济发展方式的攻坚时期。展望“十二五”，机遇与挑战并存，希望与困难同在。世界经济政治格局正在发生新的变化，国际产业结构升级和科学技术发展将带来巨大潜力和广阔空间。国内经济长期向好的基本面没有改变，环渤海地区孕育着新的发展机遇，总体环境有利于我们又好又快发展。经过长期努力奋斗，我市在产业发展、城乡建设和社会民生等方面打下了坚实基础，具备了实现新突破、再上新台阶的条件和实力。同时，我们也十分清醒地认识到，国内外经济形势依然错综复杂，国际金融危机影响深远，国内经济社会发展仍面临一些矛盾和问题，区域竞争也日趋激烈。我们要进一步增强机遇意识和忧患意识，牢牢抓住并用好重要战略机遇，适应国内外形势的新变化，顺应全市人民过上美好生活的新期待，以全球视野和战略思维，科学定位、精心谋划未来发展，更加奋发有为地推进改革开放和现代化建设。

今后五年，我市经济社会发展的指导思想是：高举中国特色社会主义伟大旗帜，以邓小平理论和“三个代表”重要思想为指导，深入贯彻落实科学发展观，以科学发展为主题，以加快转变经济发展方式为主线，以改革开放和自主创新为动力，以保障和改善民生为根本出发点和落脚点，以建设现代化国际性港口城市为目标，加快经济结构战略性调整、社会建设整体性推进、体制改革实质性突破、开放水平全方位提升，建成更高水平的全面小康社会，率先基本实现现代化，向着全面建设文明富庶新烟台的宏伟目标奋勇前进。

（一）努力建设蓝色经济强市。紧紧抓住“三大战略”机遇，以蓝色经济区建设为总抓手，坚持海陆统筹、以港兴市，向海洋进军、向内陆辐射，培育海洋、临海、涉海优势产业，形成特色化、高端化的滨海产业集群，确立国内蓝色经济领军城市地位。加快发展方式转变和产业结构调整，推动制造业由大变强、服务业跨越发展、现代农业稳步提升、战略性新兴产业和高新技术产业迅速崛起，构筑结构优化、技术先进、清洁安全、附加值高的现代产业体系。发展绿色低碳经济，狠抓节能减排，建设资源节约型、环境友好型城市，节约能源资源和保护生态环境的增长方式与消费模式初步形成。继续保持经济平稳较快增长，主要经济指标增幅高于全省和全国首批14个沿海开放城市平均水平，经济增长质量和效益明显提高。到2015年，全市生产总值达到7600亿元，年均增长12%，三次产业比例调整为5:55:40；地方财政收入达到480亿元，年均增长15%；固定资产投资年均增长16%；社会消费品零售总额年均增长16%；海洋产业产值达到2660亿元，年均增长16%；万元GDP能耗下降17%，化学需氧量、氨氮排放量均下降12%，二氧化硫和氮氧化物排放量均下降18%。

（二）努力建设创新活力城市。以更大的力度推进各领域改革，建立充满活力、富有效率、更加开放、有利于科学发展的体制机制。实施更加积极的开放战略，强化开放引领，坚持“引进来”与“走出去”并举、利用外资与国内合作并重，进一步优化开放型经济结构，增强国际竞争力。实施科教兴烟和人才强市战略，倡导创新风尚，加大创新投入，培养创新人才，建设创新平台，基本建成国家创新型示范城市。实施信息化强市战略，加快经济社会各领域信息化进程，努力打造智慧型城市。到2015年，全市外贸进出口总额突破700亿美元，实际使用外资五年累计突破60亿美元；高新技术产业产值占规模以上工业总产值的比重提高到48%。

（三）努力建设特色文化名城。坚持社会主义先进文化前进方向，推动文化大发展大繁荣，提升城市魅力和文化软实力，全面展现全国文明城市风采。加快发展文化事业，在全省率先建成覆盖城乡、布局合理、功能完善的文化设施服务网络，不断满足人民群众日益增长的精神文化需求。大力发展文化产业，形成结构优化、富有特色、竞争力较强的文化产业集群，使文化产业成为我市新的支柱性产业。充分挖掘我市特色文化，着力打造以葡萄酒文化、开埠文化、红色文化、仙道文化、莱国文化、胶东民俗文化为历史底蕴，以“创新、拼搏、协作、包容”为精神内涵，以“山海仙境?葡萄酒城”为城市形象标志，具有独特魅力的烟台文化，创建国家历史文化名城。

（四）努力建设和谐幸福家园。实施主体功能区战略，统筹城镇、产业、生态分布，做大做强中心城市，发展南北沿海两线产业走廊，保护中部生态涵养区，建设贯通南北、连接东西的立体交通网络。加快中心城市六区融合，推动县域城市向现代化、生态化、特色化、集约化方向发展，增强小城镇公共服务和居住功能，提升新农村建设水平,高标准打造精品海岸、生态海岛、美丽河畔、绿色山林，构建以中心城市为龙头、县域城市为骨干、小城镇为支撑、广大农村为腹地、路网林网海岸河道为纽带的新型城乡体系。坚持以人为本和富民优先，加快发展各项社会事业，着力改善和保障民生，积极创建和谐稳定模范城市，扎实推进社会管理创新，使城乡居民生活水平和质量持续提升，社会保障制度全面覆盖，公共服务体系更加完善，广大群众的安全感、幸福感和满意度不断提高。到2015年，中心城市人口达到200万人以上，全市城镇化水平达到63%左右；城市居民人均可支配收入和农民人均纯收入年增长均为12%；城镇登记失业率控制在3.5%以内。

各位代表！烟台的发展已经站在新的历史起点上。未来五年，全市人民将共同书写科学发展的新篇章。第三届亚洲沙滩运动会将隆重举行，烟台将以更加文明开放的形象向世界展示自己的魅力；潮水国际机场、高速铁路、烟台港西港区将投入使用，以“陆海空”立体交通为特色的东北亚交通物流枢纽城市地位将初步确立；我市将培育出5个销售收入过千亿元的产品集群，100万辆汽车、550万载重吨船舶、900万台电脑、3500万部手机的年产能将全部实现。经过“十二五”的努力，全市人均生产总值将突破15000美元，城乡居民人均储蓄将达到6万元，地方财政收入将在2010年的基础上再翻一番。我们已经踏上新的奋斗征程，勤劳勇敢的烟台人民将以自己的智慧和汗水，创造明天的更大辉煌！

三、2011年政府工作安排

2011年是中国共产党成立90周年，也是实施“十二五”规划的开局之年，做好今年经济社会发展各项工作，意义十分重大。我们将牢牢把握科学发展这个主题和转变经济发展方式这条主线，坚持以经济结构战略性调整为主攻方向，把工业化作为拉动区域发展的基本动力，把服务业作为产业结构优化升级的战略重点，把蓝色经济作为拓展新空间、打造新优势的战略支撑，提高发展的全面性、协调性和可持续性，坚持在发展中促转变，在转变中谋发展。今年经济社会发展的主要预期目标是：全市生产总值增长12%；地方财政收入增长15%；全社会固定资产投资增长16%；社会消费品零售总额增长17%；实际使用外资增长10%，外贸进出口总额增长11%；城市居民人均可支配收入和农民人均纯收入均增长12%；城镇登记失业率控制在3.5%以内；人口自然增长率控制在2.2‰以内；节能减排工作完成省下达目标。

（一）贯彻落实宏观调控政策，保持经济平稳较快发展。

着力扩大消费需求。以增强居民消费预期和消费能力为重点，完善收入分配制度，建立健全企业工资正常增长机制，努力实现居民收入增长与经济发展同步、劳动报酬增长与劳动生产率提高同步，明显增加中低收入居民的收入，加大财政用于改善民生和社会事业的投入。用足用好“家电下乡”和“以旧换新”等促进消费政策，拓展城乡消费市场。合理引导汽车、住房等大宗商品消费，推动消费结构优化升级。激活夜间经济，培育休闲旅游、文化娱乐、家政服务、体育健身等新型消费业态和新兴消费热点。继续实施“万村千乡市场”工程，加快农产品质量安全示范区与大型连锁超市的“区超对接”，畅通城乡商品流通渠道。发展节能环保型消费品，倡导文明、节约、绿色、低碳消费模式。年内，完成社会消费品零售总额1615亿元。

促进投资稳定增长。抓好总投资5966亿元的191个市级重点项目建设，完成年度投资1173亿元。发挥产业政策导向作用，引导投资向民生和社会事业、农业农村、科技创新、生态环保和资源节约等领域倾斜。以优化投资结构为重点，突出抓好烟台综合物流园区等36个商贸服务项目、市妇幼保健院等15个社会事业项目、航天高新技术产业园等19个科技创新项目。加大项目储备力度，论证筹划一批对转方式调结构具有引领作用、对区域发展具有支撑作用的重大项目。鼓励扩大民间投资,放宽市场准入,支持民间资本进入基础产业、基础设施、市政公用事业、社会事业、金融服务等领域。严格执行投资项目用地、节能、环保、安全等准入标准，规范投资行为，提高投资质量。

深入实施“三大战略”。围绕蓝色经济区建设，大力发展海洋经济和临港产业，加快建设芝罘湾港区三突堤集装箱码头、烟台港西港区和沿海港口群，高标准开发龙口人工岛群和丁字湾、莱州湾等重点区域，着力打造国家级核电基地、海洋新材料产业基地、综合性海洋装备制造业基地和现代远洋渔业基地。全年实现海洋产业产值1470亿元，增长16%。围绕高端产业聚集区建设，按照“高端、高质、高效”的要求，改造提升传统产业，引进国际前沿产业和产业前沿项目，努力构建国内一流、国际先进、优势集中、特色突出的高端产业体系。围绕黄河三角洲高效生态经济区建设，全面推动莱州及周边区域升级发展，辐射带动全市生态型经济建设。

抓好财政金融工作。认真执行积极的财政政策和稳健的货币政策,争取中央投资项目和国家、省各类扶持资金。加强财源建设，强化税收征管和非税收入管理，促进财政收入与国民经济协调增长。优化支出结构，严格控制一般性开支。保持信贷总量合理增长，加大对重点项目、重点产业、中小企业、“三农”和社会民生等领域的信贷支持。以加快金融改革创新、优化金融生态环境为重点，加强金融市场体系建设，提高金融对经济的服务能力和贡献率。探索建立创业投资引导基金、股权投资基金，年内争取发展创业投资企业15家以上。鼓励符合条件的县市区发行城投类债券，鼓励企业在境内外市场实施资本并购、上市融资和发行债券。组建烟台农村商业银行，发展村镇银行，争取更多的境内外金融机构在我市设立分支机构，支持小额贷款、信托、保险、证券、金融租赁、期货等金融业务发展。

加强市场和物价管理。正确处理好保持经济平稳较快发展、调整经济结构和管理通胀预期的关系，落实好国家稳定价格总水平的政策措施。加强地方粮油储备，确保粮食安全。坚持“菜篮子”市长负责制，搞好农副产品储运组织调度，保障群众基本生活需要。健全价格预警机制，引导生产者、经营者合理安排生产经营活动，严厉打击囤积居奇、哄抬物价、恶性炒作等违法行为，维护市场秩序。完善社会救助和保障标准与物价上涨挂钩的联动机制，保障低收入群众生活水平不因物价上涨而降低。

（二）调整振兴工业经济，推动烟台制造向烟台创造快速跃升。

大力发展先进制造业。深入实施八个行业调整振兴规划，加快提升机械制造、电子信息、食品加工、现代化工等优势特色产业，快速膨胀新能源、新材料、生物技术、节能环保、新一代信息技术等战略性新兴产业，大力发展汽车、电脑、手机、船舶及海洋工程、葡萄酒、黄金、新型铝材、核电装备、聚氨酯、新型化纤等10个产品集群，培育上下游产品，延伸产业链条，建设富有竞争力的先进制造业基地。重点抓好化学工业园、张裕工业园、通用东岳新增30万辆轿车和35万台发动机、大宇造船整船、1200万套子午胎等优势产业项目，推进海阳核电、山东国际生物科技园、东方蓝天商用飞机钛合金紧固件、瀚霖生物等新兴产业项目，探索发展低空空域通用航空业，积极引进新能源汽车等高端项目。年内，全市规模以上工业主营业务收入增长15%左右。

促进企业做大做强。启动新一轮培强做大工程，重点培育20户领军企业、50户重点企业和100户成长性企业，力争主营业务收入过百亿元企业达到13户，过10亿元企业125户以上。扶持中小企业和民营经济发展，积极构建融资担保、信用评价等中小企业公共服务体系，全面实施百家企业升级计划、千家中小企业成长计划、万家小企业培育计划和创新计划。继续深化品牌战略，加大品牌培育、宣传和保护力度，重振“北极星”和“三环”两大传统品牌，力争再创一批驰名商标和名牌产品。

积极推进科技创新。着力推动创新型城市试点工作，实施科技创新“六个十”工程。鼓励企业加大科技投入，集中抓好100个重点技术改造项目和100个重点技术创新项目，全市完成技改投入1080亿元。加强创新平台建设，搞好产学研结合，密切与国内外高等院校、科研院所的合作，组建一批以企业为主体的产业技术创新战略联盟。实施知识产权战略，加强国家知识产权示范城市建设，提高知识产权创造、管理、运用和保护水平。实施高端人才引进“双百计划”，建立完善45个高技能人才培养基地。全年新建省级以上企业技术中心6家、工程技术研究中心或重点实验室6家。全市高新技术产业产值占规模以上工业总产值的比重达到45%。

全面加强信息化建设。推动信息化与工业化深度融合，深化信息技术在产业发展、社会管理和城市建设中的应用，突出发展物联网、电子商务和高端软件业。加强重要信息系统建设，进一步完善电子政务网络。加快信息化与工业化“两化”融合试验区建设，着力打造信息化强市。

狠抓节能减排。树立绿色、低碳发展理念，提高全民节能环保意识，强化节能减排目标责任考核，防止能源消耗不合理增长。加强110户重点用能企业节能管理，抓好建筑、交通运输等重点领域节能工作，高度重视公共机构节能管理，推进节能环保工程建设，积极推广节能技术和节能产品。大力发展循环经济，加快再生资源回收体系建设，实现工业固体废弃物再利用850万吨。实施工程减排、结构减排和管理减排，严格控制化学需氧量、氨氮、二氧化硫和氮氧化物排放，加快推进南郊污水处理厂、套子湾污水处理厂二期等5个污水处理项目和大型燃煤电厂脱硫脱硝工程。

（三）优先发展现代服务业，增强对转方式调结构的带动作用。

打造产业亮点。推动旅游业大发展。充分发挥中国最佳休闲城市的品牌效应，精心打造中心城市、北部蓬莱长岛龙口、南部海阳莱阳栖霞、西部招远莱州四个旅游板块，高水平包装策划海上游、生态游、历史文化游、都市乡村游等优质旅游线路和景点景区。以滨海一线景区为纽带，抓好芝罘岛、崆峒岛、养马岛旅游开发和渔人码头等项目建设，开通游船和滨海沿线旅游观光巴士，建设国际邮轮停靠港、游艇码头等旅游设施，打响“慢游烟台”、“夜游海湾”品牌，建设中国最佳国际滨海旅游目的地。加快发展现代物流业。增辟欧美远洋集装箱航线和国际航空货运航线，扶持发展远洋运输，抓好各类物流园区建设，吸引国内外知名物流企业进驻烟台。年内，全市港口货物吞吐量达到2.3亿吨，集装箱吞吐量达到160万标箱。繁荣发展商贸餐饮业。搞好三站市场新区等大型批发市场建设，集中培育滨海广场步行街、鲁菜美食街和雨岱山海鲜美食街。促进房地产业健康发展。落实国家房地产调控政策，吸引知名地产商参与开发大型城市综合体项目。提升会展业规模和档次。办好第五届国际葡萄酒节和第十二届国际果蔬?食品博览会等会展活动，全年举办展会120个以上。膨胀发展服务外包产业。建设烟台服务外包示范园区，引进国内外知名外包企业。加大对科技信息、金融保险、商务服务、文化创意、养生养老、社区服务等新兴服务业的培植力度。年内，服务业增加值占GDP的比重达到34.5%。

加强载体建设。按照突出特色、膨胀规模、打造品牌、提升层次的要求，培育重点城区、重点园区、特色街区、50个重点企业和50个重点项目“五大服务业载体”，形成一批在国内、省内有较强影响力的重点区域、亮点行业、骨干企业和重大项目，促进全市服务经济扩规模上档次。

加大政策扶持。制定完善促进服务业发展的规划和政策，探索适合新型服务业态发展的市场管理办法，扩大市级服务业引导资金规模，研究制定规费、土地、融资等方面的倾斜政策，加大对服务业重点载体的支持力度。引导企业实施主辅业务剥离，推动企业内部服务向专业化、社会化、市场化转变，促进生产性服务业发展。

（四）扎实推进新农村建设，夯实农业农村发展基础。

积极发展现代农业。稳定粮食播种面积，建设高标准粮田，改造中低产田，提高粮食综合生产能力。优化农业产业结构，扩大高效经济作物和海珍品种养面积，加大精品蔬菜和水果基地建设力度，发展生态果业和畜禽生态养殖。完善农业科技创新和技术推广体系，大力发展现代苗种业，加强农机示范工程建设。推动农业产业化经营和标准化生产，扶持农业产业化龙头企业，鼓励发展农民专业合作社，推进“国家级绿色农业示范区”建设。开展地理标志保护产品认证和无公害、有机、绿色“三品”认证，创建更多的名牌农产品，推动农产品以更大规模、更高质量进城出境。年内，全市肉蛋奶总产量达到205万吨，水产品产量184万吨。新增农产品地理标志证明商标3件，“三品”认证110个以上。

改善农村生产生活条件。加强水利基础设施建设，加快推进胶东引黄调水工程和南水北调东线续建配套工程，开展老岚水库前期工作，完成各类水利工程3000项以上，发展节水灌溉工程18万亩。做好库区和移民安置区后期扶持工作。实施新一轮病险水库除险加固工程，争取将100座小型水库列入国家相关规划。推进农村生活污水和垃圾集中处理。新增农村沼气用户2万户，整修新建农机大院50个。

多渠道增加农民收入。全面落实强农惠农政策，确保各类涉农补贴发放到位。发展农村二三产业，引导农产品加工业在产区布局。实施新型农民培训工程，鼓励农民自主创业，促进农村富余劳动力转移就业，改善进城务工农民的就业环境，增加农民工资性收入。抓好以果品为重点的农产品促销活动，增加农民经营性收入。

（五）加大改革攻坚力度，建立健全保障科学发展的体制机制。

以完善社会主义市场经济体制为主攻方向，着力推进重点领域和关键环节改革，为加快转变经济发展方式提供强大动力。深化国有企业改革，健全国有资产监管体制，优化国有企业资本结构和治理结构，支持企业并购重组。深化财政改革，健全完善公共财政管理体系。深化农村综合改革，稳定和完善农村基本经营制度，规范土地承包经营权流转市场，加快农村集体林权制度改革，做好政策性农业保险试点工作，探索建立商业化农业保险新机制。深化医药卫生体制改革，实现基本药物制度全覆盖，所有政府办基层医疗卫生机构全部配备和使用基本药物并实行零差率销售，着力推进公立医院改革试点。继续推进行政管理体制改革，进一步巩固政府机构改革成果，稳步实施事业单位分类改革、乡镇机构改革和中介组织改革，完善市场经济主体，规范政府行政行为。

（六）实施更加积极的开放战略，提升开放型经济发展水平。

保持对外贸易平稳增长。积极转变外贸发展方式，加快培育以技术、品牌、质量、服务为核心竞争力的对外经贸新优势。鼓励加工贸易企业引进先进设备、开展技术创新、加强产品研发、延伸产业链条，引导企业争创国家级、省级科技兴贸出口创新基地。扩大机电产品和高新技术产品出口，推进旅游、信息、文化等领域的服务贸易出口，大力培育新兴出口品牌。加强出口农产品、工业产品质量安全示范区建设，提高出口产品整体质量安全水平。集中培育出口100强和进口30强企业，支持中小企业开展网上贸易。鼓励企业设立境外营销网络，巩固传统市场，拓展欧美高端市场和东盟新兴市场。落实鼓励进口政策，引导企业扩大先进技术、关键设备、节能环保产品和紧缺资源的进口。年内，全市实现外贸进出口总额486亿美元，其中出口283亿美元。

优化利用外资结构。继续开展高层招商活动，加大“三个引进”力度，强化与国内外500强企业及行业龙头、跨国公司和高等院校、科研院所的战略合作，鼓励现有外资企业增资扩股。积极开展产业招商，加强与日韩的产业互动，拓展与东盟及港澳台地区的合作领域，加大对欧美高端市场的招商力度。制定金融保险、现代物流、文化旅游等十个领域招商引资优惠政策，推进总部经济基地建设，着力引进研发中心、营销中心、采购中心等功能性机构。年内，全市实际使用外资达到12.6亿美元。加强国内经济合作，不断扩大利用内资成果。

深入实施“走出去”战略。鼓励企业积极走出国门开展经济合作，重点推进铝土矿、铅锌矿、煤矿、木材、海洋渔业等一批资源合作项目。支持万华、张裕等骨干企业并购国外企业、品牌、技术和营销网络，鼓励企业开展对外承包工程和劳务合作。

促进园区发展上水平。强化经济技术开发区在对外开放中的龙头作用，推动重大产业项目建设，加快转型升级步伐，巩固提升在国家级开发区中的先进位次。高标准建设高新技术产业开发区，大力发展高端产业项目，建设产业升级先行区、科技创新核心区、改革创新实验区和城市发展样板区，打造国内一流的国际化创新型高新区。高水平建设保税港区，发挥政策优势，提高承载功能，着力引进大型物流企业，尽快构筑出口加工、港口作业和保税物流联动发展的新格局。推动各省级园区提档升级，促进高端产业、创新要素不断向园区聚集。

（七）加强城市规划建设与管理，积极稳妥推进城镇化发展。

做精做美中心城市。按照“滨海建城、沿河开发、依山布局”的思路，加强城市总体规划编制修订工作，完善控制性详细规划和各专项规划，推动城市深度开发、品牌塑造和亮点打造，树立国际性滨海生态宜居城市新形象。“滨海建城”，重点改造提升烟台山至东炮台滨海景区和雨岱山至马山寨黄金海岸线景观，加大芝罘湾、套子湾改造力度，启动胜利、慎礼、白石等区片改造工程。高起点实施东部新城开发，重点推进龙湖前海商务区、高新区科技中央商务区等综合商务区及配套设施、景区景点建设，打造以休闲度假、科技商贸、高档商住为主要功能的现代化新城区。“沿河开发”，重点推进夹河、逛荡河、辛安河、鱼鸟河、沁水河等流域水环境治理和景观带建设，打造绿色生态的城市滨河新景区。“依山布局”，按照“透山透绿”的原则，依山就势规划建设城市新组团，推进大南山生态园、磁山地质公园、青龙山文化园等生态旅游和商住区项目，搞好山体保护性开发和破损山体恢复性治理，使城市与自然融为一体、相得益彰。精细化管理城市，年内实现六区数字化管理全覆盖。加强环境卫生综合整治，推进毓璜顶公园西扩等绿地公园建设，打造新的亮化景观组团，提升城市“洁绿亮美”水平。坚持公交优先，加强城市交通管理，健全公共交通体系，多策并举缓解交通拥堵状况。

增强城市综合承载能力。加快建设潮水国际机场、龙烟铁路、青烟威荣城际铁路，完成烟台至海阳高速公路和莱州港区疏港高速公路主体工程，确保烟台港西港区疏港高速公路和海即大桥竣工通车。启动建设长岛南北长山大桥，推进龙口港区疏港高速公路和成龙线、黄水线改建等工程，规划建设城市轨道交通、龙青高速公路。加快实施华电国际莱州电厂、百年电力四期、八角电厂等大型电力项目。加大城市道路建设力度，完善机场路、通世路等主干道立交桥，新建续建轸大路、通林路、海河西路等20余条城区道路。提升城市配套设施功能，推进城区雨污分流等工程，抓好生活垃圾处理及供热、燃气、环卫等配套设施建设。

加强县域城市和镇村建设。科学规划县域城市布局，完善城市功能，丰富城市内涵，提升城市形象。北部沿海一线，蓬莱、龙口、招远、莱州要着力塑造港口城市、现代工业新城和滨海旅游城市形象，长岛要加快建设国际休闲度假岛；南部沿海一线，海阳要以举办亚沙会为契机打造新型滨海城市，莱阳要搞好南海新区建设；中部地区，栖霞要建设烟台生态型“后花园”。扎实推进镇村建设。建好国家重点镇和省级中心镇，集中扶持市级重点镇，培育发展温泉小镇、葡萄酒小镇等一批特色镇。按照政府统筹、政策引导、群众自愿、市场运作的原则，有步骤推进农村住房建设和危房改造，全面改善广大农民的居住生活条件。

注重保护生态环境。加强重点污染源环境安全监管，建立健全空气质量监管联防联动机制，加大对建筑施工、交通运输等扬尘污染防治力度，强化机动车环保标志管理，推动公交车和出租车“油改气”。加快26个水源地保护工程建设和20条河流综合治理，搞好农村面源污染防治，保障群众饮用水安全。加强海洋生态整治和资源修复工作，建立完善覆盖全市的海洋环境监测和预报减灾体系，推进长岛国家级海洋综合气象监测实验基地建设。建好管好昆嵛山国家级自然保护区，实施三年水系造林绿化工程，年内新增造林补植面积13.5万亩，综合治理水土流失面积120平方公里。

（八）建立健全基本公共服务体系，繁荣发展各项社会事业。

实施更加积极的就业政策。坚持自主择业、市场调节就业和政府促进就业相结合，认真落实就业扶持政策，多渠道开发就业岗位，促进城乡劳动者充分就业。加强外来务工人员就业服务，促进外来务工人员在城镇稳定就业和自主创业。年内，新增城镇就业12万人以上，其中失业职工再就业3万人以上，农村劳动力转移就业2万人以上。争创国家级创业型城市，创业培训6000人以上。建立健全企业工资支付监控制度和工资保证金制度，保障劳动者权益，构建和谐劳动关系。

健全社会保障体系。继续提高企业退休人员基本养老金和老年农民养老待遇。实现新型农保全覆盖，各级财政投入6.1亿元，为全市所有60周岁以上老年农民每人每月发放基础养老金55元。调整完善城乡医保政策，提高新型农村合作医疗和城镇居民基本医疗保险政府补助标准，实现城镇基本医疗保险市级统筹。加大保障性安居工程建设力度，建立公共租赁住房制度，年内新建和筹集各类保障性住房1万套以上。健全养老服务体系，新建10处社区居家养老服务中心。大力发展慈善事业，继续开展“爱心捐助”活动。完善城乡居民最低生活保障制度，全面推行困难群众“一站式”医疗救助，健全孤儿救助保障机制，解决好困难群众就医、上学、生活等方面的实际困难。

重视发展教育体育事业。积极发展学前教育，巩固提高义务教育质量，提升高中阶段教育水平，支持驻烟高校做大做强，深化职业教育集团化办学。科学合理调整学校布局，基本完成中小学校舍安全工程建设任务，优化教师队伍和教育资源配置，促进教育均衡发展。加强校安工程建设，提高学校、幼儿园安全保卫水平。深入贯彻实施《全民健身条例》，广泛开展全民健身活动，不断提高竞技体育水平。搞好场馆设施建设和赛事组织，全力做好第三届亚沙会筹备工作。

加快医疗卫生事业发展。提高基本公共卫生服务经费人均标准，健全疾病预防控制等专业公共卫生服务网络，严防严控重点传染病。调整优化卫生资源布局，完善基层医疗卫生服务体系，抓好县级医院、乡镇卫生院和社区卫生服务中心建设。加大公共场所、学校卫生和医疗市场的监管力度，确保公共卫生安全。建立医疗纠纷调解机制，保障医患双方合法权益。加强卫生应急体系和120急救网络建设，增强重大突发事件卫生应急处置能力。坚持中西医并重，支持中医药事业发展。

繁荣发展文化事业和文化产业。加强社会主义核心价值体系建设，全面实施道德建设工程，广泛开展群众性精神文明创建活动，争创第三批全国文明城市。围绕庆祝建党90周年、纪念辛亥革命100周年，开展形式多样的文化活动，丰富群众文化生活。加快建设五彩文化广场、世界葡萄酒文化博览园、中青产业创意园和古登州文化大观园等项目，繁荣发展新闻出版、传媒展览等文化产业，培育壮大骨干文化企业，推动文化产业提升发展。加强县市区文化馆、图书馆、乡镇综合文化站和农家书屋建设，搞好广播电视村村通工程。

推动其他社会事业全面发展。做好人口与计划生育工作，稳定低生育水平，提高生育保险待遇标准。积极发展老龄、妇女、儿童、残疾人和红十字事业，抓好外事侨务、气象地震、民族宗教、史志档案和仲裁工作，加强国防教育、人民防空、民兵预备役和双拥共建工作，扎实开展对口支援工作。

（九）加强和创新社会管理，进一步巩固和谐稳定的良好局面。

做好信访稳定工作，健全社会稳定风险评估和大调解机制，从源头上预防和减少矛盾。坚持不懈抓好安全生产，全面落实安全生产责任制，促进安全生产形势继续稳定好转。强化食品药品监管，有效防控食品药品安全风险。实施政府放心粮油工程，保障群众基本饮食安全。加强防灾减灾能力建设，完善各类突发事件应急预案，规划建设应急指挥信息系统。强化社区管理，做好流动人口服务管理、特殊人群帮教管理，提高城乡社区自治和服务功能。进一步扩大基层民主，稳步推进第十届村委会换届选举工作，规范村务公开民主管理。深入推进和谐稳定模范城市建设，加强社会治安综合治理，抓好社区警务、群防群治等基础建设，严密防范、依法打击各类违法犯罪活动。开展法治烟台创建活动，启动“六五”普法依法治理工作，加强法律宣传、法律服务和法律援助。探索建立以“惠民、安民、便民、乐民”为目标的社会管理和服务体系，努力开创全市人民各尽其能、各得其所，心情舒畅、和睦相处的良好局面！

四、加强政府自身建设

（一）提升思想境界，科学把握形势，增强驾驭复杂局面的本领。牢固树立科学、和谐、率先发展理念，进一步解放思想、更新观念，在全国、全省大格局中确立高目标、谋划新思路、推动大发展，促进各个领域创新发展、转型发展、跨越发展，努力在转方式调结构的新一轮竞争中走在前面。建设学习型政府，深入学习实践科学发展观，全面提升推动科学发展的能力、应对风险挑战的能力、抢抓发展机遇的能力。加强对国内外形势的研究和全市经济运行情况的分析，借鉴国内外先进城市发展经验指导具体工作，采取科学有效的政策措施推动经济社会又好又快发展。

（二）弘扬实干精神，狠抓工作落实，提升政务运行效率和质量。建设务实政府，在各级政府机关倡导想干事、能干事、干成事的良好风气。大兴求真务实之风，发扬埋头苦干精神，说实话、办实事、求实效，坚决杜绝形式主义。坚持开展调查研究，继续加强市长公开电话、市长信箱建设，深入了解社情民意，及时解决基层、企业和群众反映集中的实际困难和问题，多干打基础、利长远的实事，多办解民忧、惠民生的好事。始终把狠抓工作落实作为政府工作的重要职责，严格执行督查制和问责制，形成一级抓一级、层层抓落实的工作局面，努力增强政府执行力。认真办理人大代表建议和政协提案，不断提高办理质量和效率。深化行政审批制度改革，进一步精简和规范审批事项，推动网上审批，加强行政审批电子监察，设立乡镇便民服务中心，提高政务服务水平。

（三）坚持依法行政，科学民主决策，不断提高政府的公信力。进一步转变政府职能，完善执法监管制度，规范行政执法行为和自由裁量权，促进行政执法人员严格执法、公正执法、文明执法。坚持向人大及其常委会报告工作和向政协通报情况制度，自觉接受人大法律监督和政协民主监督，加强与各民主党派、工商联、无党派人士的联系。健全科学民主依法决策机制，完善专家咨询论证、社会公示和听证制度，主动听取社会各界的意见建议，提高决策透明度和公众参与度。继续推进政务公开、政府信息公开，加快电子政务建设，畅通政府与群众沟通渠道。扎实开展民主评议工作，促进政风行风建设。

（四）牢记为民宗旨，保持清廉本色，树立良好的公务员队伍形象。牢固树立正确的政绩观、群众观、权力观，千方百计为群众谋福祉，努力创造经得起实践、人民和历史检验的业绩。深入实施为民服务实事工程，切实解决好人民群众最关心、最直接、最现实的利益问题。完善从政行为监督制约机制，加强财政监督和审计监督，确保行政权力在阳光下运行。深入开展节约型机关创建活动，进一步降低行政运行成本。全面落实党风廉政建设责任制，更加注重标本兼治、惩防并举，更加注重完善制度、规范权力，严格执行《廉政准则》，强化反腐倡廉教育，筑牢拒腐防变的思想道德防线，健全完善惩治和预防腐败体系，努力建设风清气正、高效廉洁、人民满意的公务员队伍。

各位代表！“十二五”规划蓝图宏伟而壮丽，新的发展征程任重而道远。让我们更加紧密地团结在以胡锦涛同志为总书记的党中央周围，高举中国特色社会主义伟大旗帜，全面落实科学发展观，在中共烟台市委的坚强领导下，万众一心、奋发进取，开拓创新、扎实工作，为全面完成“十二五”规划目标而努力奋斗！
